# Trauma-focused treatment for traumatic stress symptoms in unaccompanied refugee minors: a multiple baseline case series

**DOI:** 10.3389/fpsyg.2023.1125740

**Published:** 2023-06-01

**Authors:** Carlijn Maria Van Es, Merel E. Velu, Marieke Sleijpen, Niels van der Aa, Paul A. Boelen, Trudy Mooren

**Affiliations:** ^1^Department of Clinical Psychology, Utrecht University, Utrecht, Netherlands; ^2^ARQ Centrum’45, ARQ National Psychotrauma Centre, Diemen, Netherlands

**Keywords:** unaccompanied refugee minors, trauma-focused treatment approach, multiple baseline, mixed-methods, posttraumatic stress disorder, depression

## Abstract

**Introduction:**

Unaccompanied refugee minors (URMs) are at increased risk of developing mental health problems, such as symptoms of posttraumatic stress disorder (PTSD) and depression. In addition, URMs face several barriers to mental health care. Few studies have evaluated trauma-focused interventions for URMs that target these issues. The current study evaluated a multimodal trauma-focused treatment approach for URMs. It aimed to provide an initial indication of the effectiveness of this treatment approach and to provide a qualitative evaluation assessing treatment satisfaction of the participating URMs.

**Methods:**

A mixed-methods study was conducted among ten URMs, combining quantitative data with qualitative data through triangulation. Quantitative data were collected using a non-concurrent multiple baseline design in which repeated, weekly assessments were carried out during a randomized baseline period, during treatment, and during a 4-week follow-up period. Questionnaires assessing PTSD (Children’s Revised Impact of Event Scale) and symptoms of depression (The Patient Health Questionnaire-9, modified for adolescents) were used. In addition, treatment satisfaction was measured post-treatment using a semi-structured interview.

**Results:**

During the qualitative evaluation, all but one URM noted they found the trauma-focused treatment approach useful and felt the treatment had positively impacted their wellbeing. However, the results of the quantitative evaluation did not show clinically reliable symptom reductions at posttest or follow-up. Implications for clinical practice and research are discussed.

**Discussion:**

The current study presents our search in developing a treatment approach for URMs. It adds to the current knowledge about methodological considerations in evaluating treatments for URMs, the potential effects of trauma-focused treatments on URMs, and the implementation of treatments for URMs.

**Clinical trial registration:** The study was registered in the Netherlands Trial Register (NL8519), 10 April 2020.

## 1. Introduction

In 2019, approximately 40% of all refugees in the world were minors (The UN Refugee Agency, 2020). About 7% of the minors applying for asylum in the European Union that year arrived without a primary caregiver (European Migration Network, 2020). These unaccompanied refugee minors (URMs) are more vulnerable to develop psychological complaints than accompanied minors arriving in the country of resettlement with a parent or primary caregiver (Huemer et al., 2009; Vervliet et al., 2014a,b). For example, Derluyn and Broekaert (2008) found that URMs in Belgium were five times more likely to develop severe symptoms of anxiety, depression, and posttraumatic stress disorder (PTSD) than accompanied refugee minors. (Comorbid) PTSD, depression, and anxiety are the most prevalent disorders amongst URMs in Europe (Bamford et al., 2021; Daniel-Calveras et al., 2022). The increased vulnerability to developing mental health problems is assumed to be due to several risk factors, including the separation from their parents, a high exposure to potentially traumatic events, and the loss of their familiar environment and support system, whilst being faced with the continuous stressors associated with migration (McKelvey and Webb, 1995; Derluyn and Broekaert, 2008; Demazure et al., 2018).

URMs in the Netherlands go through the same asylum procedure as adult asylum seekers, but have additional rights to education and guidance. URMs get appointed a guardian by Nidos, a family guardian organization. They receive free housing and education. In addition, URMs without asylum status receive free health care and URMs with asylum status receive health care insurance. URMs in the Netherlands report several daily stressors, including worries about legal status, family reunification, and finances (van Es et al., 2019).

Research shows that certain trauma-focused interventions, including trauma-focused cognitive behavioral therapy (TF-CBT) and Eye Movement Desensitization and Reprocessing (EMDR) adequately address trauma-related complaints in traumatized children without flight experiences (Rodenburg et al., 2009; Goldbeck et al., 2016). In addition, TF-CBT, Narrative Exposure Therapy for children (KIDNET), and EMDR are proven to be promising treatments for traumatized refugee children (Oras et al., 2004; Ehntholt et al., 2005; Onyut et al., 2005; Wadaa et al., 2010; Said and King, 2020; Velu et al., 2022). However, little is known about optimal approaches to diminish distress in URMs (Unterhitzenberger et al., 2015; Demazure et al., 2018). Few studies on psychotherapeutic interventions addressing the specific problems and challenges URMs encounter have been performed. The majority of these studies is solely qualitative or based on case descriptions (Demazure et al., 2018; Unterhitzenberger et al., 2019). This gap in knowledge on interventions might, in part, be due to the barriers faced when offering interventions to URMs. For example, URMs are often preoccupied with continuous daily stressors, such as worries about the wellbeing of their family members and a complex family reunification procedure (Nickerson et al., 2011; van Es et al., 2019). Other barriers include difficulties in establishing a trusting relationship with adults, linguistic and cultural differences, and poor access to services (Bean et al., 2006; Derluyn and Broekaert, 2008; Ni Raghallaigh, 2013; Demazure et al., 2018). As a result, URMs may not receive the help they need. Diminishing these barriers and offering culturally sensitive and accessible interventions to this group of minors are key public health challenges (Ehntholt and Yule, 2006).

To overcome the aforementioned barriers, a culturally-sensitive, multimodal trauma-focused treatment approach specifically for URMs in The Netherlands was developed (Van Es et al., 2021). The multimodal trauma-focused treatment approach aims to diminish and overcome the aforementioned barriers and to address the specific, individual needs of traumatized URMs. The approach is described in more detail in (Van Es et al., 2021). Although the approach is trauma-focused, URMs do not have to talk extensively about their traumatic experiences which may help to overcome reluctance to disclose negative experiences. The therapist collaborates with an Intercultural Mediator (ICM) prior to and during each session to further reduce cultural and language difficulties. ICMs are close to the URMs in cultural background and experience. They aim to facilitate communication between the therapist and the URM, as they interpret language and offer knowledge on the cultural background of the URMs. Collaborative work with an ICM is assumed to help in building a trusting relationship and making interventions culturally sensitive. Finally, care is offered at or near the living environment of URMs to allow them to receive the intervention in a familiar environment, to save them the effort of traveling, and to prevent them from feeling different from others because they have to go to a mental health institution. Moreover, as the URMs do not have any travel time, they do not miss so many school hours.

Based on the findings of a feasibility trial (Van Es et al., 2021), it was suggested that the approach partly overcomes barriers to mental health care. To further evaluate the treatment approach, we designed the present study, using a mixed methods, non-concurrent multiple baseline design with ten participants with elevated symptoms of depression and/or PTSD. Although randomized controlled trials (RCTs) are considered the golden standard when evaluating the effectiveness of a program, Demazure et al. (2018) stated that the feasibility of conducting an RCT with URMs is limited. Therefore, Demazure et al. (2018) propose using alternative methods, including small-N designs. An advantage of multiple baseline designs is that they require smaller samples than an RCT, as statistical power is generated by within subject evaluation and participants serve as their own control. A multiple baseline design allows us to distinguish the effect of treatment from that of time and allows for more causal interpretations than an open trial (Arntz et al., 2013; Renner et al., 2016). The aims of the current study were: (1) to provide an initial indication of the effectiveness of this multimodal trauma-focused approach for traumatized URMs and (2) to provide a qualitative evaluation assessing treatment satisfaction of the participating URMs. As this is one of the first studies to examine the effectiveness of this treatment, this study can also inform future research efforts on how to conduct research among URMs.

## 2. Materials and methods

### 2.1. Procedure

This study was a collaboration between ARQ Centrum’45 (a specialized mental health care institute for the treatment of complex psychotrauma complaints) and Nidos (a guardianship institution for unaccompanied and separated children under the age of 18). ICMs were employed by Nidos and guardians were informed about the trauma-focused treatment approach by youth care professionals working at (Nidos). Nidos guardians who observed symptoms of PTSD and/or depression among URMs and barriers to regular mental health care were informed of the possibility to refer URMs to ARQ Centrum’45, in consultation with the minors. All patients who were consecutively referred to ARQ Centrum’45 for the trauma-focused treatment approach between June 2019 and December 2020 and who received the trauma-focused treatment from one of the participating therapists were invited to take part in the study. This time period is equivalent to the study period of the 10 participants. The intervention took 10.5 weeks on average. Because of the COVID-pandemic the study was paused between February 2020 and November 2020 as outreach care could not be offered.

Guardians were informed about the study *via* telephone. Subsequently, the URMs were invited for an intake. A therapist, a researcher from ARQ Centrum’45, an ICM, and -in most instances- the guardian were present during the intake interview. In addition to the intake interview, the researcher and the ICM offered the URMs verbal information about the nature of the study, its purpose, procedures, expected duration, and the possible benefits and risks involved in participation. An information letter and informed consent form were handed out to the URM and, if necessary, translated by the ICM. URMs signed the written informed consent form. For URMs under the age of 16, the legal guardian also signed the written informed consent. The first 10 consecutive eligible participants who agreed to take part in the study were included in the present study.

In this non-concurrent multiple baseline study, we randomized participants over five different baseline (waitlist) periods of 4, 5, 6, 7, and 8 weeks, respectively. A random sequence of 10 different baseline periods was generated using the software package Random Allocation Software (Random Allocation Software, 2004) by an independent researcher NA. The sequence was generated such that each baseline period appeared twice in the sequence. The independent researcher was contacted in order to obtain the baseline period once a new participant was included in the study. During the baseline period, participants did not undergo any intervention.

During this study, the first and last assessments of the URMs were conducted by an independent researcher MV with the help from an ICM. Information on demographic variables and requests for help were collected during the intake interview. Questionnaires measuring symptoms of PTSD (Children’s Revised Impact of Event Scale; CRIES-13) and symptoms of depression (The Patient Health Questionnaire-9, modified for adolescents; PHQ-A) were administered weekly during the baseline period, treatment period, and a 4-week follow-up period. These measurements were conducted *via* the telephone by the ICMs. In addition, during this phone call, the ICM asked the following questions: (1) How are you doing? and (2) Do you have any questions? Finally, after the 4-week follow-up period participants were invited for an individual interview conducted by a researcher and ICM to evaluate the trauma-focused treatment. The study was approved by the Medical Ethical Committee of ARQ Centrum’45.

#### 2.1.1. Therapists

The trauma-focused treatment was offered by therapists working at ARQ Centrum’45. Therapists were licensed mental health care workers and trained EMDR and NET-therapists, with multiple years of experience working with refugee minors from different cultural backgrounds. Therapists took part in a one-day training, multidisciplinary consultation, and supervision offered by the study center ARQ Centrum’45. Supervision was organized in team meetings where cases, based on self-reports of therapists, were discussed. Therapists discussed which modules would suit which minor and request for help during multidisciplinary consultation and supervision.

#### 2.1.2. Participants

Ten consecutive patients referred to the trauma-focused treatment approach by their legal guardian or general practitioner were included in this study. Participants were URMs with elevated symptoms of PTSD and/or depression, living in the Netherlands, referred to ARQ Centrum’45, and who received treatment from one of the four participating therapists. In order to be eligible to participate in this study, participants had to meet all of the following criteria: (1) being a URM under the guardianship of Nidos, (2) aged up to 19 (as some URMs may receive extended youth care after turning 18, minors up to 19 years old could be referred for treatment), (3) presenting symptoms of PTSD and/or depression based on psychological evaluation, and (4) with consent to participate in the study from the URM and her/his guardian. Potential participants meeting any of the following criteria were excluded from participation in this study: (1) acute suicidality, (2) acute psychosis, and (3) if there was a need to consult or involve a psychiatrist, for example, when medication or crisis intervention was required. Clinicians checked the criteria based on information from the referral and/or intake interview.

**Figure 1:**
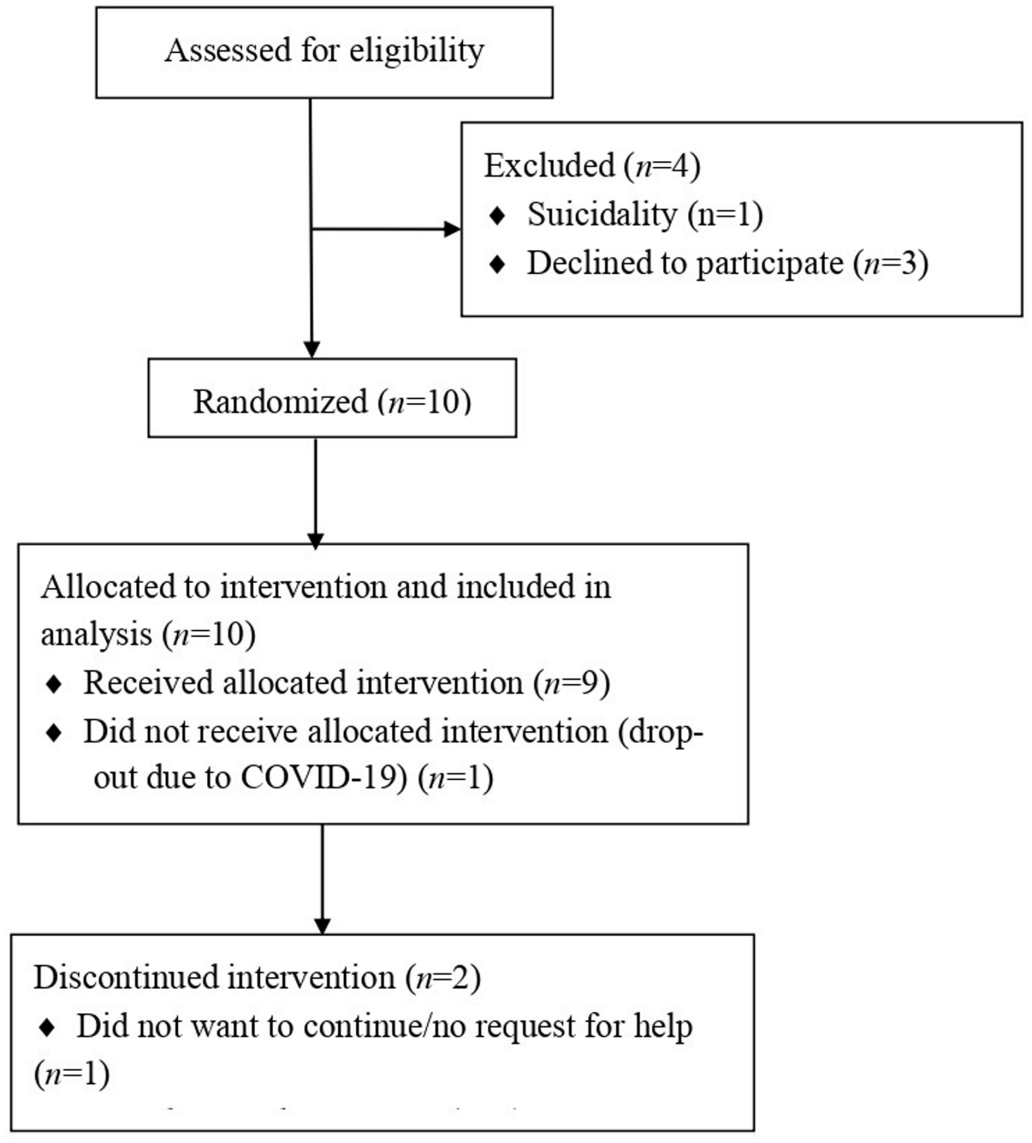
Flowchart of participants.

The flowchart can be found in Figure 1. Three participants prematurely terminated treatment. One participant dropped out because outreach care could not be offered because of restrictions due to COVID-19. One participant heard their asylum application was refused and moved to another country. Lastly, one participant reported no complaints after a few sessions and stated she wanted to stop treatment to focus on her daily life.

#### 2.1.3. Treatment approach

The treatment approach consists of approximately eight, 80-min face-to-face sessions targeting ongoing stressors and symptoms of depression and (traumatic) stress. During the first session, a clinical intake interview takes place. In the following sessions, psychoeducation is offered and the treatment rationale is explained. Then, the URMs’ lifeline (as derived from KIDNET) is laid out. Next, it is decided during multidisciplinary consultation which treatment modules suited the request for help of the URMs best. The multimodal approach includes cognitive behavioral interventions, i.e., KIDNET, EMDR, activation, and cognitive restructuring and exposure. Treatment modules were chosen based on the request for help and complaints reported by the minor. The approach and the procedure is described in more detail in (Van Es et al., 2021).

### 2.2. Instruments

#### 2.2.1. Symptoms of posttraumatic stress

The CRIES-13 (Children and War Foundation, 1998) was administered to measure posttraumatic stress symptoms. The CRIES-13 is available in several languages (www.childrenandwar.org). The questionnaires were translated to Arabic and Tigrinya by ICMs prior to initiation of the study. The 13-item scale includes three subscales: intrusion, avoidance, and arousal. The subscales are based on the Impact of Events Scale (IES; Horowitz et al., 1979) and the DSM-IV. Items are rated on a 4-point scale with anchors 0 = *not at all,* 1 = *rarely,* 3 = *sometimes,* and 5 = *often* with a time period of the past seven days. A PTSD score is calculated by summing all item scores. In line with Verlinden et al. (2015), data from the CRIES-13 were counted as missing if more than one item on a subscale was missing. If a maximum of one item per subscale was missing on the CRIES-13, missing values were replaced by the mean of the completed items on the same subscale, following the method presented by Kocalevent et al. (2013). Higher scores indicate more severe PTSD symptoms. A score ≥ 30 suggests an increased risk of PTSD (Perrin et al., 2005). Good psychometric properties have been reported for the CRIES-13 and it has been used extensively among children exposed to war and with different cultural backgrounds (Smith et al., 2003; Perrin et al., 2005; Verlinden et al., 2014).

#### 2.2.2. Symptoms of depression

To measure symptoms of depression, the PHQ-A (the Patient Health Questionnaire-9, modified for adolescents; Johnson et al., 2002) was used. This measure is adapted from the Patient Health Questionnaire-9. The PHQ-A is available in English and was translated to Arabic and Tigrinya by an ICM prior to initiation of the study. It includes nine items rated on a 4-point scale ranging from 0 (*not at all*) to 3 (*nearly every day*) with a time period of the past 7 days. A total score was computed by summing all individual item scores with higher scores reflecting more severe depressive symptoms. In line with prior studies on the PHQ-9 (Kroenke et al., 2010), data were counted as missing if more than two items were missing. If two or less items were missing on the PHQ-A, missing values were replaced by the mean of the completed items (Kocalevent et al., 2013). Following research on the PHQ-9, a total score ≥ 10 was considered as the cut-off score for detecting depression (Manea et al., 2012). Despite a few differences, the PHQ-9 is mostly consistent with DSM-5 criteria for a major depressive disorder (Kroenke et al., 2001). The PHQ-9 is widely used in studies among adolescents and good psychometric properties have been reported (Kroenke et al., 2001; Richardson et al., 2010). Although psychometric properties of the PHQ-A for refugee minors from different (cultural) backgrounds have, to our knowledge, not yet been studied, the PHQ-A has been shown to have acceptable psychometric properties when completed by Arabic refugee minors (Al-Amer et al., 2020).

#### 2.2.3. Evaluation of treatment

Finally, a semi-structured qualitative interview was conducted by an independent researcher at the follow-up to qualitatively evaluate the treatment and assess treatment satisfaction according to the URMs. The qualitative interview consisted of questions about usefulness (“Was the treatment helpful, and if so, in what way?”; “Is there anything else you need?”), (emotional) change (“If you look back upon how you were feeling/functioning before you received this therapy and how you are doing now – what are the biggest changes?”), satisfaction (“What did you like/dislike about the treatment?”; “Would you recommend the treatment to someone with similar experiences?”), and questions concerning specific treatment components (“What did you think of the number of sessions?”; “Would you have minded traveling to receive treatment sessions?”).

### 2.3. Statistical analysis

#### 2.3.1. Quantitative analysis

First, visual inspection of the data was carried out in order to provide insight in the individual course of PTSD and depressive symptom severity during the baseline, intervention, and post-intervention period. Weekly obtained assessment data of the CRIES-13 and PHQ-A collected during the baseline, intervention, and post-intervention period were plotted in separate graphs for each participant. In order to establish whether observed intraindividual changes in PTSD and depressive symptom severity reflected statistically reliable changes, the Reliable Change Index (RCI) procedure as described by Jacobson and Truax (1992), was used. The RCI is calculated as the ratio between the difference between two test scores obtained at two measurement occasions and the standard error of the difference score (SED). The SED was calculated based on baseline standard deviations derived from the study sample and test–retest reliability coefficients (0.85 and 0.84 for the CRIES-13 and PHQ-9, respectively) reported by studies on the psychometric properties regarding the CRIES-13 and PHQ-9 (Kroenke et al., 2001; Verlinden et al., 2014). Baseline standard deviations in the current sample were comparable to those reported in studies on psychometric properties of the PHQ-A and CRIES-13 (Kroenke et al., 2001; van der Kooij et al., 2013; Verlinden et al., 2014). RCI values larger than 1.96 (or smaller than −1.96) indicate that there is a statistical reliable intraindividual difference between two test scores, i.e., with 95% certainty, the difference between the test scores is due to actual change (improvement or deterioration) rather than measurement error. RCIs were calculated for the difference in PTSD and depressive symptom severity during baseline (t_1_−t_2_), treatment (t_2_−t_3_), and follow-up (t_3_−t_4_). t_1_ refers to the first baseline assessment, t_2_ to the last baseline assessment, t_3_ to the first follow-up assessment, and t_4_ to the last follow-up assessment.

Missing data points were left out of the visual graphs. For the RCI of the CRIES-13, respectively one, two, one, and one data points were missing for baseline, pre-treatment, post-treatment and follow-up. For the RCI of the PHQ-A, respectively none, none, one, and one data point was missing for baseline, pre-treatment, post-treatment, and follow-up. Missing data points for the RCI were handled using next observation carried backwards/forwards. Specifically, missing baseline data points were imputed using the first available data point from the baseline period. Missing data points during the pre-treatment period were imputed using the last available data point from the baseline period. If post-treatment data points were missing, these were imputed using the first available data point from the follow-up period. If follow-up data points were missing, these were imputed using the last available data point from the follow-up period.

#### 2.3.2. Qualitative analysis

Minutes taken during the qualitative evaluation interviews were analyzed using MAXQDA 10 (VERBI). The data were then analyzed using the General Inductive Approach (Thomas, 2003). In this approach, data analysis is guided by the evaluation objectives. First, the texts were read thoroughly. Second, specific text fragments that were linked to the research questions were identified. Third, fragments were labelled to create categories. These steps were conducted independently by two researchers CE And MV. During the fourth step, the overlap and redundancy of the categories were reduced. Finally, the most important categories were described. Both researchers discussed the categories until they reached a consensus. These five steps resulted in outcome categories that represented the most important themes.

#### 2.3.3. Integrating data

We conducted a non-concurrent mixed methods study, using a triangulation design (Creswell and Plano Clark, 2011). The aim of this design is to improve our understanding of a specific topic by obtaining complementary data. Using this design, quantitative and qualitative data are collected simultaneously. After data collection, one researcher CE combined, compared, and contrasted the quantitative results and qualitative results. The integrated results are presented, describing whether the qualitative and quantitative data resulted in similar findings as well as highlighting different findings.

## 3. Results

The average age of participants was 16.5 (SD = 1.08; range 15–18) years. Nine participants (90%) came from Eritrea and one from Syria (10%). Two participants were female (20%). Table 1 summarizes participant’s main problems, the main focus of the sessions, no-show/drop-out, and additional comments. Missing data points of participants A, F, and G were due to drop-out. Other missing data points were mostly related to a participant (C) not being able to get out of bed; a participant (E) who experienced too much stress concerning family reunification to continue with the assessments; and a participant (H) did not want to continue with the questionnaires as she found it took too much time.

**Table 1:** Treatment overview per participant.

Participant	Number of sessions	Main problem/request for help	Treatment module	No-show/drop-out	Comments	Qualitative/Quantitative Results
A	4	Forgetfulness, sleeping, nightmares	Intake, lifeline, and psychoeducation	Drop-out: did not want to continue, no request for help and too many daily stressors	Difficulties with foster mother.	*Quantitative.* Decrease in symptoms of depression during baseline. *Qualitative.* Did not take part in the interview.
B	11	Feeling down/insecure	Intake, lifeline, EMDR	Cancelled twice because of school activities and father passing away	Father passed away during treatment.	*Quantitative.* Decrease in symptoms of depression during baseline, increase in symptoms of depression during treatment, no change from start of treatment to follow-up. *Qualitative.* Increased focus, self-care, being proud of themselves talking to other about the past.
C	9	Feeling tired	Intake, lifeline	-	Received news that his father was not his biological father; is lying in bed for days; worries about asylum status; physical complaints; focus on establishing social network and activation.	*Quantitative.* A decrease in symptoms of depression during baseline and treatment, an increase in symptoms of depression during follow-up, no change from start of treatment to follow-up. *Qualitative.* Less physical aches, came out of bed more often, started remembering appointments.
D	7	Questions concerning identity, worries about physical health	Intake, lifeline, EMDR	-	Stress concerning relationship with family; worries about physical health and family reunification; questions concerning identity.	*Quantitative.* An increase in symptoms of depression from start treatment to follow-up, no other changes. *Qualitative.* Improved relationship with loved ones, still experiencing worries about family reunification
E	7	Sleeping, concentration, stress	Intake, lifeline, EMDR	-	Worries about family reunification; discussed sleeping hygiene.	*Quantitative.* An increase in symptoms of PTSD during baseline, a decrease in symptoms of PTSD during treatment, and an increase in symptoms of PTSD during follow-up, no change from start treatment to follow-up. An increase in symptoms of depression during baseline, follow-up and from start treatment to follow-up. *Qualitative.* An improvement in sleep and concentration, new problems in Eritrea and in school after treatment caused feelings of depression.
F	0	Feeling down, suicidal thoughts	Intake	No-show twice during intake, drop-out after moving country	Declared age of majority/illegal; moved to another country.	*Quantitative.* No changes during baseline. *Qualitative.* Did not take part in the interview.
G	0	Sleeping, concentration, overthinking	-	Drop-out because of COVID-19	-	*Quantitative.* No changes during baseline. *Qualitative.* Did not take part in the interview.
H	8	Stress, sleeping, avoiding contacts because of memories	Intake, lifeline, EMDR	-	Worries about family and friends in Eritrea; positive news concerning family reunification.	*Quantitative.* A decrease in symptoms of depression and PTSD during baseline. No change from start treatment to follow-up. *Qualitative.* Thinking more about positive memories, feeling ‘less bad’ about the negative memories.
I	9	Sleeping, feeling more calm, concentration	Intake, lifeline, EMDR	Cancelled twice because of quarantine due to COVID-19	Bad news concerning family reunification; difficulties with peers.	*Quantitative.* A decrease in symptoms of depression during baseline, no other changes. *Qualitative.* Forgot negative memories, no other effects, prefers focusing on the future.
J	9	Stress and difficulties sleeping	Intake, lifeline	-	Worries about family members and family reunification.	*Quantitative.* A decrease in symptoms of depression and PTSD, no other changes. *Qualitative.* The treatment helped with practical issues, more keen to go to appointments

EMDR, Eye Movement Desensitization and Reprocessing; PTSD, posttraumatic stress disorder.

### 3.1. Symptoms of PTSD and depression

**Figure 2:**
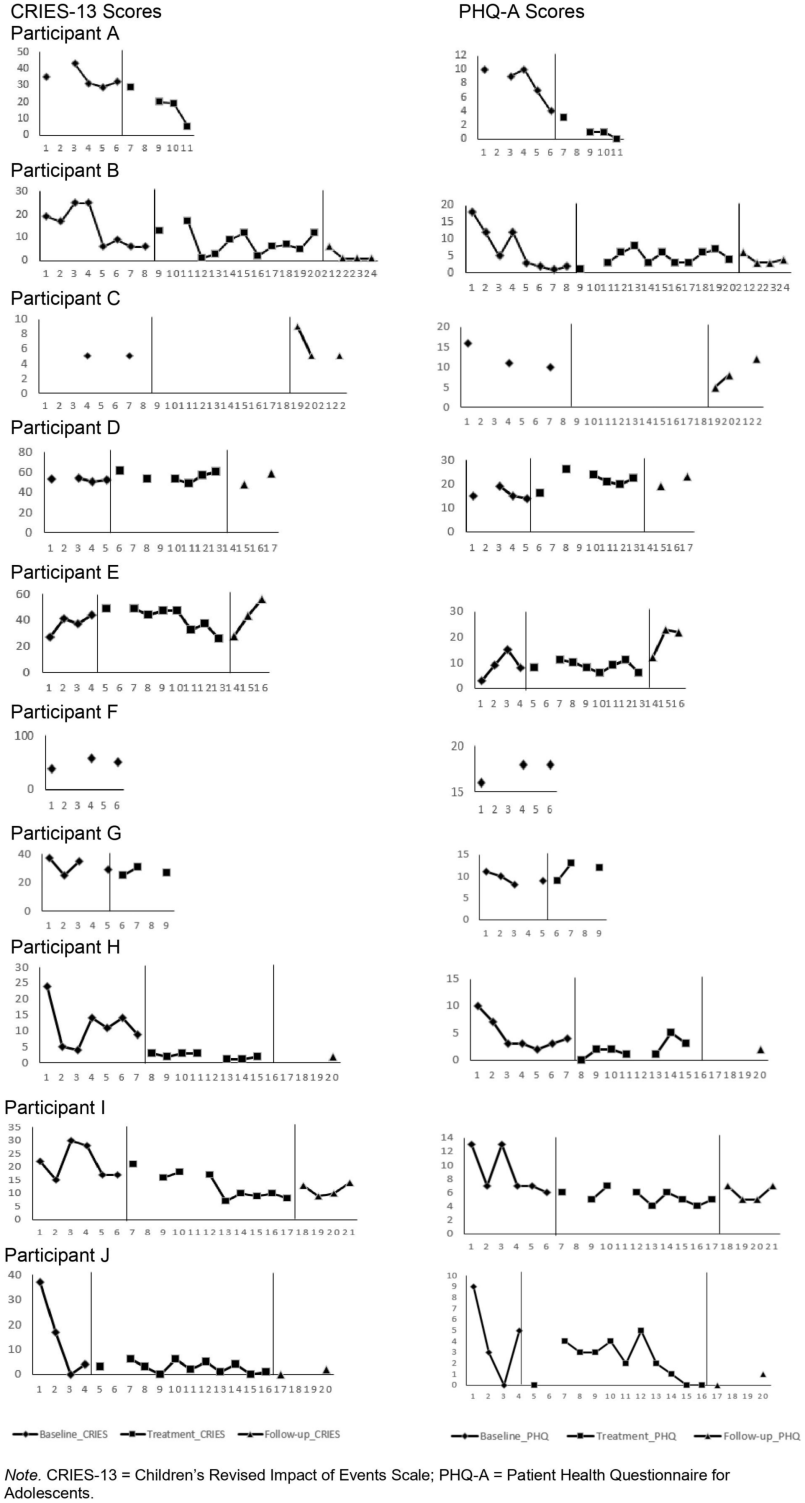
Individual scores on the PHQ-A and CRIES-13 over time. CRIES-13 = Children’s Revised Impact of Events Scales; PHQ-A = Patient Health Questionnaire for Adolescents.

The baseline, pre-treatment, post-treatment, and follow-up measures are presented in Table 2. Weekly assessments of PTSD and depressive symptom severity are presented in Figure 2. Visual inspection suggests a decrease in symptoms of depression during the baseline period, but negligible change during treatment and follow-up. Moreover, fluctuations in symptoms can be seen in several participants during the baseline period (e.g., in participant B, I, and J).

**Table 2:** Baseline, pre-treatment, post-treatment, and follow-up measurements.

Measure	Baseline			Pre-treatment			Post-treatment			Follow-up		
	*n*	M	SD	*n*	M	SD	*n*	M	SD	*n*	M	SD
CRIES-13	10	29.8	13.3	9	23.2	20.6	6	17.3	17.6	7	19.9	26.2
PHQ-A	10	12.2	4.4	10	7.1	6.4	6	8.2	6.6	7	10.1	9.2

CRIES-13, Children’s Revised Impact of Event Scale; PHQ-A, The Patient Health Questionnaire-9, modified for adolescents.

Table 3 presents the RCI scores during baseline (t_1_−t_2_), treatment (t_2_−t_3_), follow-up (t_3_−t_4_), and between the start of treatment and end of follow-up (t_2_−t_4_). During the baseline period, two participants improved with regard to PTSD symptom severity, one worsened, and six remained unchanged. During treatment, only one participant improved with regard to PTSD symptom severity. However, this participant deteriorated during follow-up and overall, remained unchanged. The other participants (*n* = 5) remained unchanged with regard to PTSD symptom severity during treatment. Overall, none of the participants evidenced statistically significant changes from the beginning of treatment to follow-up.

**Table 3:** RCI of symptoms of PTSD and depression.

Participant	RCI t_1_−t_2_ CRIES-13	RCI t_2_−t_3_ CRIES-13	RCI t_3_−t_4_ CRIES-13	RCI t_2_−t_4_ CRIES-13	RCI t_1_−t_2_ PHQ-A	RCI t_2_−t_3_ PHQ-A	RCI t_3_−t_4_ PHQ-A	RCI t_2_−t_4_ PHQ-A
A	−0.83	–	–	–	−2.79^*^	–	–	–
B	−0.83	−0.96	−0.69	−1.65	−6.77^*^	1.99^*^	−0.80	1.20
C	0.00	0.55	−0.55	0.00	−2.39^*^	−1.99^*^	2.79^*^	0.80
D	1.10	−1.83	1.56	−0.28	0.40	1.25	1.54	2.79^*^
E	3.03^*^	−2.87^*^	3.87^*^	1.01	1.99^*^	1.59	3.98^*^	5.58^*^
F	1.65	–	–	–	0.80	–	–	–
G	−1.65	–	–	–	−0.80	–	–	–
H	−2.89^*^	–	–	−0.14	−3.98^*^	–	–	0.80
I	−0.14	−1.10	0.14	−0.96	−2.79^*^	0.40	0.00	0.40
J	−4.68^*^	−0.41	0.28	−0.14	−3.59^*^	0.00	0.40	0.40

* Reliable change in symptoms.

CRIES-13, Children’s Revised Impact of Event Scale; PHQ-A, The Patient Health Questionnaire-9, modified for adolescents; PTSD, posttraumatic stress disorder; RCI, Reliable Change Index; t_1_, first baseline assessment; t_2_, last baseline assessment; t_3_, first follow-up assessment; t_4_, last follow-up assessment. Negative numbers refer to decreases in symptoms, whereas positive numbers refer to increases in symptoms.

Six participants improved with regard to depressive symptom severity during baseline, one deteriorated, and three remained unchanged. During treatment, one improved, one worsened, and four remained unchanged. During follow-up, two deteriorated, and four remained unchanged. From start of treatment to follow-up two worsened and five remained unchanged.

### 3.2. Evaluation by participants

Participants who completed the study protocol from baseline to follow-up (*n* = 7) took part in the qualitative evaluation of the multimodal trauma-focused treatment. Most participants (*n* = 6) found the treatment helpful and would recommend it to others. They appreciated the outreach work, as five participants stated they would not have participated if they had to travel to a mental health institution. Moreover, three participants valued the occasional informal, personal nature of the contact. Some explained that they appreciated that the ICM sometimes stayed after a session to talk, walk, or cook together. For example, one URM said drinking coffee and walking with the ICM helped her after a difficult session, explaining: “[*After coffee*] *I walked home as a new person.*” Four participants reported that a useful aspect of the treatment was talking about their experiences with the therapists and ICMs. URMs explained they felt free to share their experiences, were relieved after talking, and felt space to discuss subjects they would not address with others. One URM added:

“They come back, and again, they don’t give up. I’m starting to think: can I share my problems with these people? And then I started sharing. […] Sometimes I was tired and wanted to sleep and did not want to talk, but they came back and helped me and little by little, I started talking.”

Finally, two participants stated they found the lifeline helpful. One added:

“Now I have a chance to see all different parts of my life, the good and the bad. […] This offers me balance.”

All but one participant noticed an impact of the treatment. For example, some (*n* = 3) noted they could address difficult topics with loved ones and experienced an improved relationship with friends and family. Other benefits included an improved ability to concentrate, feeling proud of themselves, improved self-care, and experiencing improved sleep. The one URM who did not notice an impact explained that although negative experiences did not bother him so much anymore, the treatment did not impact his life. He would rather focus on his future, such as his education or social network. Another URM stated:

“The biggest difference between before and after? Before I wasn’t interested, I didn’t feel like doing anything. I didn’t want to go to school. Or didn’t go to appointments. Sleeping was also difficult. After and during the treatment, I feel like I’m more keen, I go to school and to the appointments.”

Notably, most URMs (*n* = 5) spoke about the daily stressors they continued to experience, including worries about the future, anxiety concerning family reunification, troubles with peers, and worries about the lives and wellbeing of family members. For example, the father of a URM passed away, another URM received the news that his father was not his biological father, and yet another URM was declared illegal during the treatment. In addition, most URMs came from Eritrea, and during this study there was turmoil in their country of origin, leaving them worried about the lives of their families. Some explained that their complaints increased due to these issues.

“Now there is a new problem concerning school and my family in Eritrea. […] Right after the treatment I had less difficulties sleeping and concentrating. So now, after this situation I have nightmares and I am sleepwalking again. Because of these problems I’m depressed. Sometimes I contemplate suicide. I have a lot of problems now.”

### 3.3. Integrating qualitative and quantitative data

In the qualitative evaluation, all but one URM noted they found the treatment useful and felt the treatment had positively impacted their wellbeing. However, this impact was not visible in the quantitative evaluation. The qualitative evaluation included results, such as improved sleep and improved ability to concentrate, that would be expected to manifest in the quantitative evaluation. However, other qualitative findings, including being able to address difficult topics with loved ones, are not expected to be reflected directly in the questionnaires measuring PTSD and depression. For example, participant B reported no change from start of treatment to follow-up on symptoms of depression and PTSD, but did report increased self-care and being proud of herself.

The impact of continuous stressors was sometimes reflected in both the quantitative as well as the qualitative data. For example, participant E reported an increase in symptoms of PTSD and depression during follow-up. The qualitative data indicated that this might have resulted from new problems that occurred at school and the tumultuous circumstances in Eritrea, where his family resided. Notably, most changes in symptoms of PTSD and depression were observed during the baseline period. No qualitative data on this time period were collected.

## 4. Discussion

The current study explored the potential effectiveness and treatment satisfaction of a trauma-focused treatment approach in a small sample of URMs, suffering from symptoms of PTSD and/or depression. In addition, we aimed to provide implications for future research on how to conduct research among URMs. Although URMs are among the most vulnerable groups of refugees, studies evaluating the effectiveness of trauma-focused treatments for URMs remain scarce. We expected to find a decrease in symptoms of PTSD and depression considering that our modular treatment included promising treatment interventions, including EMDR and KIDNET. Notably, the baseline period was associated with a larger decrease of mental health issues than the treatment period. The results of our quantitative evaluation do not show clinically reliable symptom reductions at posttest or follow-up.

A question that arises is whether the offered treatment approach suited the current needs of the URMs. Potentially, another treatment, such as culturally adapted cognitive behavioral therapy (CBT) with attention for emotion-regulation and continuous stressors, might have been more suitable (Hinton et al., 2012). In addition, the limited number of sessions focused on EMDR or CBT might not have been enough to cause a significant change in symptoms of PTSD or depression. Another possibility is that the questionnaires do not reflect the actual impact of the treatment approach. For instance, the main problem reported by the URMs was not always related to PTSD and depression, but more often to psychosocial functioning. Consequently, the treatment approach might have focused more on, and consequently affected, the psychosocial functioning and/or quality of life of the URMs. As suggested by the qualitative findings, the treatment might have had a positive impact on the URMs that is not directly related to symptoms of PTSD and depression, as some URMs, for example, indicated that they noticed an improvement in selfcare and in the will to discuss difficult topics with loved ones.

In line with previous studies, we found an indication that the treatment effect was impacted by continuous stressors (Unterhitzenberger et al., 2019; Van Es et al., 2021). URMs were faced with a wide range of strenuous daily stressors during treatment, including the passing of a father and bad news concerning family reunification. Additionally, during the time of this study there was turmoil in the border region of Ethiopia and Eritrea, an area where many Eritrean refugees reside (British Broadcasting Corporation, 2021). This resulted in major worries concerning family reunion procedures as well as the lives and wellbeing of friends and family residing in Eritrea and Ethiopia. The impact of current stressors on mental health has been shown in several studies (Laban et al., 2005; Drožđek et al., 2014; Unterhitzenberger et al., 2019). Although we did not systematically evaluate the impact of current stressors on PTSD and depression, or the impact of mental health problems on current stressors, some URMs indeed communicated that their mental health was impacted by continuous stressors. When offering trauma-focused treatment for URMs, it is of great importance to pay attention to reducing stressors resulting from the past as well as continuous stressors. This is not only of relevance because URMs report the impact of both stressors, but also because PTSD symptoms can maintain and provoke further daily stressors and vice versa. For example, school problems can be a result of the lack of concentration and conflicts with peers can be a result of hyperarousal (Neuner et al., 2010).

All but one participant came from Eritrea. In Eritrea, talking about psychological problems is often seen as shameful and giving voice to dissatisfaction is often seen as being ungrateful. As a result, URMs taking part in the interviews may have been hesitant to answer questions about their current psychological wellbeing and satisfaction with the treatment, which may have resulted in socially desirable answers (Nidos, 2018).

Most changes in symptoms were observed during the baseline period. This suggests that the symptoms of PTSD and depression were not stable in this sample during this period of time. The changes during baseline might have been due to events in the lives of these URMs (e.g., news concerning family reunification). Another explanation might be that the weekly contact with the ICM, who conducted the questionnaires and assessed how the URM was doing, positively impacted the wellbeing of the URMs. However, as we did not systematically assess events or other factors that may have impacted the mental health of URMs during the baseline period this limits our ability to make any statements on the cause of the changes during this period. Arntz et al. (2013) suggested the multiple baseline design might be more suited for stable problems without a large time effect during the baseline period. As the baseline periods were unstable, it was more difficult to distinguish the effect of treatment from that of time. Using a longer baseline period in future studies might result in a more stable baseline.

The feasibility of the current study was influenced by factors related to the setting and population, including news concerning asylum status and family reunification. In our earlier study, we found that the feasibility of the assessments was low, possibly as a result of therapists conducting the assessments (Van Es et al., 2021). Although the involvement of ICMs in the assessments increased response rates, some URMs did not complete all questionnaires. Most participants who refused to fill in questionnaires did so because they were experiencing (too much) stress. Missing questionnaires and drop-out did not seem to be related to the nature of the treatment.

### 4.1. Strengths and limitations

Strengths concerning the study include that it is one of the first to evaluate the effectiveness of a trauma-focused treatment approach specifically for URMs. Moreover, the study is conducted in a clinical, naturalistic setting. Another strength is that, in contrast to our feasibility study (Van Es et al., 2021), the assessments were not conducted by the therapist. However, the ICM conducting the questionnaires was also involved in the treatment and both the ICM and researcher were aware of the treatment status and -condition. Finally, an important strength of the current study is the combination of quantitative and qualitative methods. This mixed methods approach has helped us in broadening our understanding of the needs of the participants as well as their experiences with the treatment approach.

Limitations include the restricted generalizability of the current findings. Firstly, all but one URM came from Eritrea. Secondly, the substantial number of drop-outs and missing data might have affected study outcomes. Thirdly, the generalizability is affected by the small sample size. Another limitation is the use of the CRIES-13 to measure symptoms of PTSD, as the questionnaire is not in line with the contemporary DSM-5 or ICD-11. Moreover, the questionnaire could possibly be filled in with a continuous stressor in mind instead of a prior traumatic event, as it was not combined with a questionnaire assessing stressors (Criterion A).

Furthermore, the questionnaires used in this study were not validated for an Eritrean population. In addition, the translations aimed to provide a direct translation of the questionnaires and did not account for cultural appropriateness of questions or translation of cultural concepts (Pernice, 1994; Robila and Akinsulure-Smith, 2012). However, a study amongst traumatized refugees in the Netherlands suggested that local idioms of distress may not play a major role when assessing PTSD, anxiety, and depression (Wind et al., 2017). Although we aimed to overcome the aforementioned challenges by collaborating with ICMs in the assessments and combining qualitative data with quantitative data, we should be aware that the background and culture of these URMs might have affected the results of the current study.

### 4.2. Scientific implications

It must be stressed that the challenges we faced during this study should not discourage future research, as these URMs deserve specialized treatment, adapted to their specific needs. The findings of this study have several possible scientific implications. First, future research is needed to broaden our understanding of the acceptability and effectiveness of the presented trauma-focused treatment approach. It was difficult to establish the effectiveness of the current treatment approach as the treatment approach was offered in a flexible manner and therapists were free to choose modules that best suited the needs of the URMs. This resulted in a wide variety of subjects addressed during the treatment sessions, again resulting in difficulties in drawing conclusions about the effectiveness of the treatment approach. We found that the treatment approach partly overcomes barriers to treatment in a highly specialized population that is not motivated for treatment. In addition, most URMs evaluated the approach positively and stated it had positively impacted their wellbeing. More research is needed to further understand which treatment components were helpful, and which components did not contribute to the acceptability and effectiveness of the treatment approach. Until further examination of this treatments is conducted, preliminary implementation is cautioned.

Second, future research efforts might focus on other promising treatments for URMs. For example, Unterhitzenberger et al. (2019) painted a promising picture, indicating that TF-CBT is feasible and possibly effective in diminishing symptoms of PTSD in URMs. In addition, a research protocol was recently published, describing a RCTs comparing stepped-care models to care as usual for URMs (Rosner et al., 2020).

One of the aims of the current study was to inform future research efforts. One important lesson learned during this study is that it is important to look beyond clinical measures of symptoms of PTSD and depression. Such assessments may not capture the potential effect of programs offered to URMs. Potentially, as suggested by the qualitative results of the current study, the strength of this treatment approach lies in lowering barriers to mental health care, building a trusting relationship, and improvements in social functioning, global functioning and quality of life. Future research efforts could use quantitative assessments measuring such aspects of functioning. In addition, the current study shows the benefit of conducting mixed methods research when working with URMs. Finally, the results of the current study suggest that it would be helpful to use resources such as outreach care and ICMs when offering treatment to URMs.

### 4.3. Conclusion

The current study represents our ongoing search in developing a suitable treatment approach for an understudied population deserving the treatment they need. This study adds to the knowledge about methodological considerations in evaluating treatments for URMs, the potential effects of trauma-focused treatments, and the implementation of treatments for URMs.

## Data availability statement

The datasets presented in this article are not readily available because of the sensitive nature of the data. Requests to access the datasets should be directed to c.van.es@arq.org.

## Ethics statement

The studies involving human participants were reviewed and approved by Medical Ethical Committee of Leiden University. Written informed consent to participate in this study was provided by the participants' legal guardian/next of kin. Written informed consent was obtained from the individual(s), and minor(s)' legal guardian/next of kin, for the publication of any potentially identifiable images or data included in this article.

## Author contributions

MV, CE, and MS coordinated the project. TM contributed to the development of the treatment protocol. CE, TM, MS, MV, and PB designed the study. CE took the lead in writing the manuscript. NA provided statistical support. All authors contributed to the article and approved the submitted version.

## Funding

This work was supported by the Asylum, Migration and Immigration Fund (AMIF) under project number 2017EFA2017. AMIF had no role in the implementation of study.

## Conflict of interest

The authors declare that the research was conducted in the absence of any commercial or financial relationships that could be construed as a potential conflict of interest.

## Publisher’s note

All claims expressed in this article are solely those of the authors and do not necessarily represent those of their affiliated organizations, or those of the publisher, the editors and the reviewers. Any product that may be evaluated in this article, or claim that may be made by its manufacturer, is not guaranteed or endorsed by the publisher.
